# Murine Cytomegalovirus Deubiquitinase Regulates Viral Chemokine Levels To Control Inflammation and Pathogenesis

**DOI:** 10.1128/mBio.01864-16

**Published:** 2017-01-17

**Authors:** Adam T. Hilterbrand, Daniel R. Boutz, Edward M. Marcotte, Jason W. Upton

**Affiliations:** a Department of Molecular Biosciences, Institute for Cellular and Molecular Biology, LaMontagne Center for Infectious Disease, University of Texas at Austin, Austin, Texas, USA; b Department of Molecular Biosciences, Institute for Cellular and Molecular Biology, Center for Systems and Synthetic Biology, University of Texas at Austin, Austin, Texas, USA; Brown University

## Abstract

Maintaining control over inflammatory processes represents a paradox for viral pathogens. Although many viruses induce host inflammatory responses to facilitate infection, control is necessary to avoid overactivation. One way is through the manipulation of proinflammatory chemokine levels, both host and viral. Murine cytomegalovirus (MCMV), a model betaherpesvirus, encodes a viral C-C chemokine, MCK2, which promotes host inflammatory responses and incorporates into virions to facilitate viral dissemination. Here, we show that the activity of M48, the conserved MCMV deubiquitinating enzyme (DUB), regulates MCK2 levels during infection. Inactivation of M48 DUB activity results in viral attenuation and exacerbates virally induced, MCK2-dependent inflammatory responses. M48 DUB activity also influences MCK2 incorporation into virions. Importantly, attenuation of DUB-mutant virus acute replication *in vitro* and *in vivo* is largely ameliorated by targeted deletion of MCK2. Thus, uncontrolled MCK2 levels appear to mediate DUB-mutant virus attenuation in specific tissues or cell types. This demonstrates that MCMV M48 DUB activity plays a previously unappreciated role in controlling MCK2 levels, thereby managing MCK2-dependent processes. These findings reveal a novel intrinsic control mechanism of virally induced inflammation and support the identification of betaherpesvirus DUBs as possible new targets for antiviral therapies.

## IMPORTANCE

Human cytomegalovirus infections represent a tremendous burden not only to those afflicted but also to health care systems worldwide. As cytomegalovirus infections are a leading cause of nongenetic sensory loss and neurodevelopmental delay, it is imperative that valuable model systems exist in order that we might understand what viral factors contribute to replication and pathogenesis. Currently, the only approved drug treatments against CMV infection are nucleoside analogues, to which some strains have become resistant. Understanding unique viral enzymatic contributions to infections will allow the development of novel pharmacological therapies. Here, we show that M48, the conserved MCMV deubiquitinase, is critical for MCMV replication in mice and demonstrate that attenuation is due to deregulated production of a viral proinflammatory chemokine. The deubiquitinases of both human and murine CMV represent structurally unique DUBs and are therefore attractive targets for pharmacological intervention. Continued research into the substrates of these DUBs will lend additional insight into their potential as targets.

## INTRODUCTION

The prototypic betaherpesvirus, human cytomegalovirus (HCMV), remains a major causative agent of nongenetic birth defects, such as sensory loss, defects in neural development, and microcephaly (1). Although immunocompetent patients typically present no obvious symptoms of infection, HCMV still represents a major cause of morbidity and mortality in immunocompromised or immunonaive individuals, including transplant recipients and infants infected prenatally (1). In order to navigate the multitude of antiviral responses in an immunocompetent host, cytomegaloviruses devote a significant portion of their genome to counteracting or appropriating host responses to facilitate successful infection (2, 3). CMV encodes a number of proteins involved in various processes, such as type I interferon (IFN) (4–6), cell death pathways (7), and major histocompatibility complex class I antigen presentation (8), that act to antagonize or appropriate antiviral responses. In addition to intracellular immune modulation, cytomegaloviruses encode cytokines and chemokines to modulate immune responses at a distance (9).

Due to the high degree of species specificity, tractable model systems are required for host/pathogen studies of the cytomegaloviruses. The use of the murine cytomegalovirus (MCMV) model system has provided significant insight into CMV biology over the years (10). HCMV and MCMV share significant genetic and biological characteristics; among them is the large tegument protein (LTP). This essential protein (11–13) encodes a structurally distinct deubiquitinase within its amino terminus (14). Though not essential for replication (15–18), the DUB activities possessed by LTPs in other herpesviruses have been ascribed a number of distinct functions.

Emerging evidence suggests that herpesvirus DUBs play roles in manipulating host antiviral responses to infection. Murine gammaherpesvirus 68 (MHV 68) and Kaposi’s sarcoma-associated herpesvirus (KSHV) DUBs can deubiquitinate RIG-I, thus removing its ability to signal to downstream components and propagate type I IFN signaling (19). The herpes simplex virus 1 (HSV-1) DUB, UL36, can target tumor necrosis factor (TNF) receptor-associated factor 3 (TRAF3), thereby blocking type I IFN production (20). The Epstein-Barr virus (EBV) DUB, BPLF1, blocks NF-κB signaling via the deubiquitination of several proteins involved in Toll-like receptor (TLR) signaling, namely, TRAF6, NEMO, and IκBα (21). Recent work also demonstrated a conserved function for herpesvirus DUBs in antagonizing stimulator of interferon gene (STING)-dependent interferon responses in bone marrow-derived dendritic cells (BMDCs) (22), further implicating herpesvirus DUBs in the modulation of antiviral signal transduction.

Additionally, several herpesvirus DUBs have been ascribed roles in pathogenesis. The DUB of pseudorabies virus (PRV) targets itself for deubiquitination, allowing transmission to nerve termini and subsequent axonal retrograde transport to facilitate neuroinvasion (23). Inactivation of the Marek’s disease virus (MDV) DUB led to a decrease in lymphoma incidences in chickens (24). Moreover, an MHV 68 DUB mutant showed rapid clearance from infected spleens (25) and a decrease in the establishment of STING-dependent latency (22). Those studies indicated that herpesvirus DUBs contribute to replication and pathogenesis in their hosts. Recent work has also shown that the HCMV DUB is active during infection, contributes modestly to replication, and maintains virion stability and infectivity (17, 26). However, *in vivo* analyses examining the contribution of CMV DUB activity to replication in a live host have not been conducted. In this report, we show that the MCMV DUB, M48, significantly contributes to the replication and dissemination of MCMV *in vivo* by regulating levels of MCK2, the virally encoded proinflammatory chemokine. In regulating MCK2 levels, aspects of MCK2 biology, namely, MCK2-dependent inflammatory responses and incorporation of MCK2 into virions, are also controlled. Interestingly, concomitant loss of MCK2 in the presence of a mutant DUB restored replication in most cell types in culture as well as in the spleen and liver of infected mice. These results highlight MCMV DUB contributions to acute infection *in vitro* and *in vivo* and provide important mechanistic insight into the role that it plays during infection in a natural host. It appears that MCK2-dependent responses are managed through DUB-dependent regulation of MCK2 levels.

## RESULTS

### MCMV DUB activity contributes modestly to replication in cell culture.

**FIG 1:**
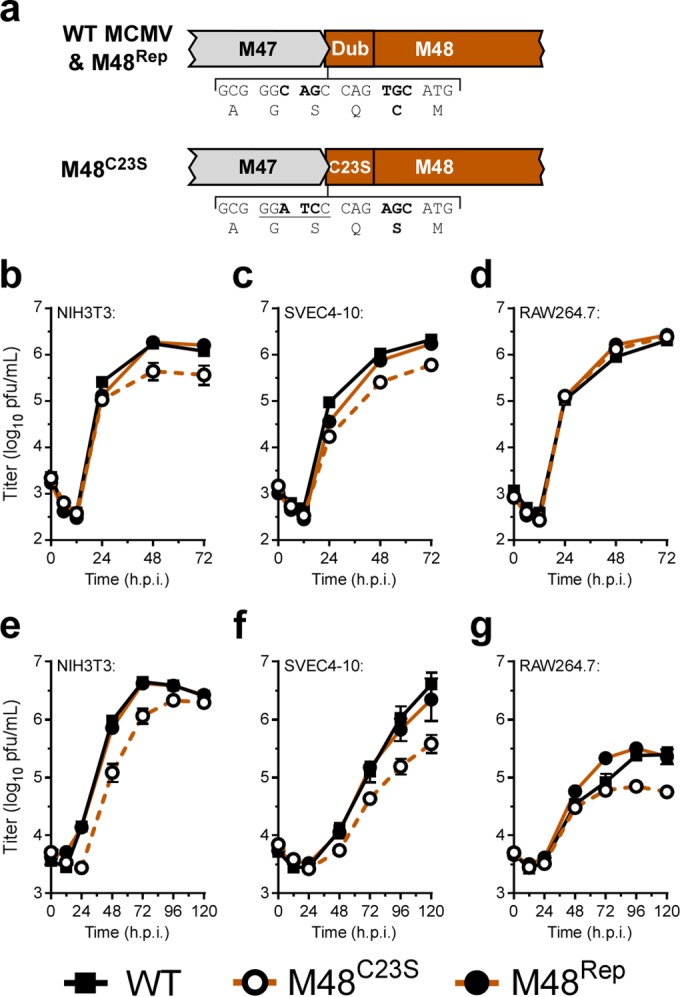
MCMV DUB activity modestly contributes to replication in cell culture. (a) Schematic of the genomic location of and mutagenesis performed in the MCMV DUB, M48. (b to g) Single-step (b to d) (MOI = 5.0) and multistep (e to g) (MOI = 0.05) growth curves in NIH3T3 fibroblasts (b and e), SVEC4-10 endothelial cells (c and f), or RAW264.7 macrophages (d and g). Each data point represents *n* of ≥6 replicates. The 0 h time point represents the time immediately postwash and addition of fresh complete media. h.p.i., hours postinfection.

In a previous study, we generated an MCMV DUB mutant (MCMV-M48^C23S^) (22). Briefly, MCMV-M48^C23S^ was generated by 2-step allelic exchange mutagenesis of the pARK25 bacterial artificial chromosome (BAC) containing the K181 Perth strain of MCMV by first inserting a selection/counterselection cassette (SacB/Kan^r^ [kanamycin resistance]) into the open reading frame of M48. The catalytic cysteine of the M48 DUB was then replaced with a serine residue (Fig. 1a; see also Fig. S1a to c in the supplemental material). This mutation was repaired to restore the wild-type (WT) sequence by allelic exchange to control for spurious distal mutations, generating the MCMV-M48^Rep^ virus, which behaved similarly to WT virus *in vivo* (Fig. S1e and f). Importantly, the C23S mutation was sufficient to inactivate M48 DUB activity (Fig. S1d). To begin the characterization of MCMV-M48^C23S^, we first sought to determine the contribution of MCMV M48 DUB activity to viral replication in culture. Single-step growth analyses of infected cells in culture revealed that MCMV-M48^C23S^ is modestly attenuated for growth in fibroblasts (Fig. 1b) and endothelial cells (Fig. 1c) compared to WT or the M48^Rep^ viruses. In contrast, mutant and control viruses replicated similarly in a macrophage cell line (Fig. 1d), suggesting a potential cell type-specific role for DUB activity during lytic replication. Multistep growth of the mutant virus displayed similar levels of attenuation in all 3 cell types (Fig. 1e to g). Together, these results reveal that M48 DUB activity plays a minor role in viral replication that is similar to the role ascribed to the DUB activity of the HCMV homologue, UL48 (17).

10.1128/mBio.01864-16.1 FIG S1  Generation of MCMV-M48^C23S^ and MCMV-M48^Rep^ recombinant viruses. (a) Schematic diagram depicting the genomic area around M48 as well as the mutagenesis strategy. Numbers represent MCMV genomic coordinates (GenBank accession number AM886412.1), and abbreviations indicate restriction enzyme sites (Hi, HindIII; Ec, EcoRI; Ba, BamHI). Using a two-step allelic exchange strategy (see Materials and Methods), a selection/counterselection cassette was introduced into the N-terminal portion of M48 and was then replaced by a sequence introducing a single amino acid substitution (C23S) and synonymous mutations introducing a unique BamHI restriction site. The nucleotides and amino acids that were changed are indicated in bold. Underlined sequences indicate an introduced restriction enzyme site. The gray bar indicates a PCR amplicon generated as described for panel C. (b) Restriction fragment length polymorphism (RFLP) analysis of WT, M48SK-1, M48^C23S^, M48SK-2, and M48^Rep^ bacterial artificial chromosomes (BAC). Isolated DNAs were digested with the *EcoRI* restriction enzyme, separated on a 0.6% agarose gel, stained with ethidium bromide, and visualized on a Typhoon Imager. (c) Infectious virion DNA from the indicated viruses was isolated, and amplicons from the M48 locus were generated by PCR. Amplicons were digested with *BamHI* (specific mutation) or *Taqα1* (present in all). Reactions were separated on 1.0% agarose gels, stained with ethidium bromide, and visualized on a Typhoon Imager. (d) Inactivation of the DUB was verified by cloning amino acids 1 to 285 from BACs (MCMV-M48^C23S^ or WT) into expression vector pEGFP-N1. NIH3T3 cells were transiently transfected with either the empty vector (EV) control or WT M48^(1–285)^-EGFP or C23S M48^(1–285)^-EGFP. Total ubiquitin levels were assessed by immunoblotting 24 h posttransfection. (e) Spleen titers of WT mice infected with BAC-derived WT (vARK25) or MCMV-M48^Rep^ 3 days postinfection with 10^6^ PFU via intraperitoneal injection. (f) Liver titers of WT mice infected with BAC-derived WT MCMV (vARK25) or MCMV-M48^Rep^ 3 days postinfection with 10^6^ PFU via intraperitoneal injection. Download FIG S1, PDF file, 0.9 MB. Copyright © 2017 Hilterbrand et al. 2017 Hilterbrand et al. This content is distributed under the terms of the Creative Commons Attribution 4.0 International license.

### MCMV DUB activity is critical for replication in mice, and a mutant DUB virus elicits a greater inflammatory response.

**FIG 2:**
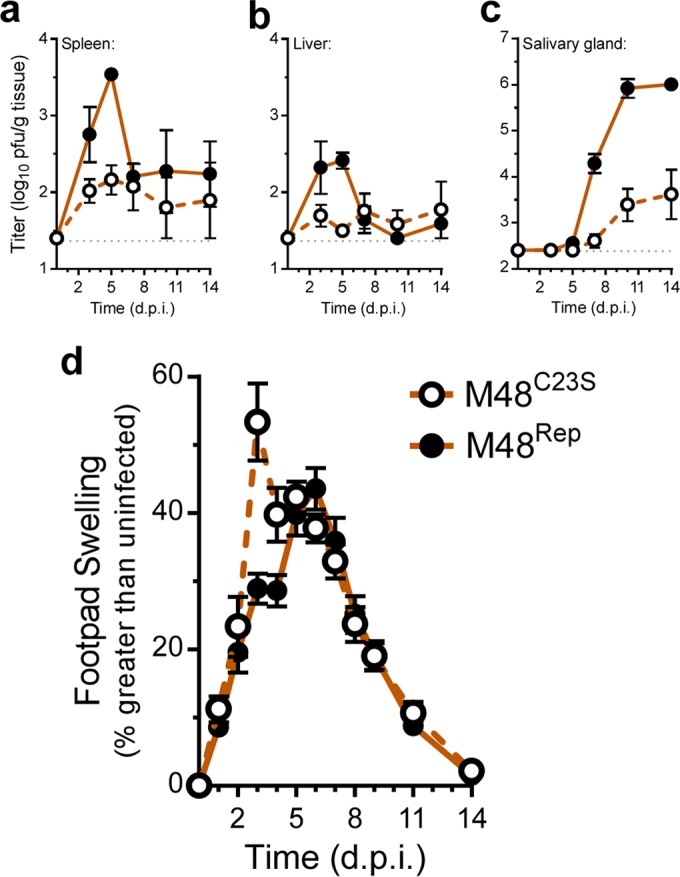
MCMV DUB activity is critical for replication in mice, and a mutant DUB virus elicits a greater inflammatory response. (a to c) Organ titers from C57BL6/J mice infected (i.p.) with 10^6^ PFU of either MCMV-M48^C23S^ or MCMV-M48^Rep^. Spleen (a), liver (b), and salivary gland (c) were collected and assessed at the indicated days postinfection (d.p.i.). Each data point represents *n* of ≥5 mice. (d) Footpad swelling of C57BL6/J mice infected with 10^6^ PFU of either MCMV-M48^C23S^ or MCMV-M48^Rep^ via footpad injection. Swelling was measured by digital caliper over the course of 14 days, and data are plotted as percent increase over the measurements from uninfected mice. Each data point represents *n* of ≥5 mice.

We next sought to determine the contributions of M48 DUB activity to replication in a natural host. C57BL/6J mice were inoculated with either MCMV-M48^C23S^ or MCMV-M48^Rep^ virus by intraperitoneal (i.p.) injection. Acute replication in spleen, liver, and salivary glands was assessed at the indicated times postinfection. Compared to the repaired virus, MCMV-M48^C23S^ was severely attenuated for acute replication in all organs assessed. Although replication of both mutant and repaired virus in the spleens of infected mice peaked at 5 days postinfection, MCMV-M48^C23S^ replication achieved peak levels 24-fold lower than those seen with MCMV-M48^Rep^ (Fig. 2a). Viral titers in infected livers (Fig. 2b) revealed that MCMV-M48^C23S^ replicated at or slightly above the limit of detection and displayed no obvious peak of replication, whereas MCMV-M48^Rep^ replicated to the expected levels in this organ (27). MCMV-M48^C23S^ was detected in the salivary glands of animals by 14 days postinfection; however, the levels were approximately 240-fold lower than those seen with MCMV-M48^Rep^ (Fig. 2c). Together, these results show that the DUB function of M48 is critical for MCMV replication in a natural host.

We continued our *in vivo* characterization of MCMV-M48^C23S^ by examining the resulting host response to the virus following footpad inoculation with MCMV-M48^C23S^ or MCMV-M48^Rep^. Footpad inoculation allows the assessment of virus-induced inflammation at the site of inoculation, characterized by swelling of the infected footpad. Surprisingly, MCMV-M48^C23S^ caused a rapid onset of swelling by 3 days postinfection not seen in the MCMV-M48^Rep^ virus-infected mice (Fig. 2d). Thus, despite being attenuated for acute replication *in vivo*, the DUB-mutant virus elicited a more robust inflammatory response.

### M48 DUB activity regulates MCK2 levels and can regulate secretion.

**FIG 3:**
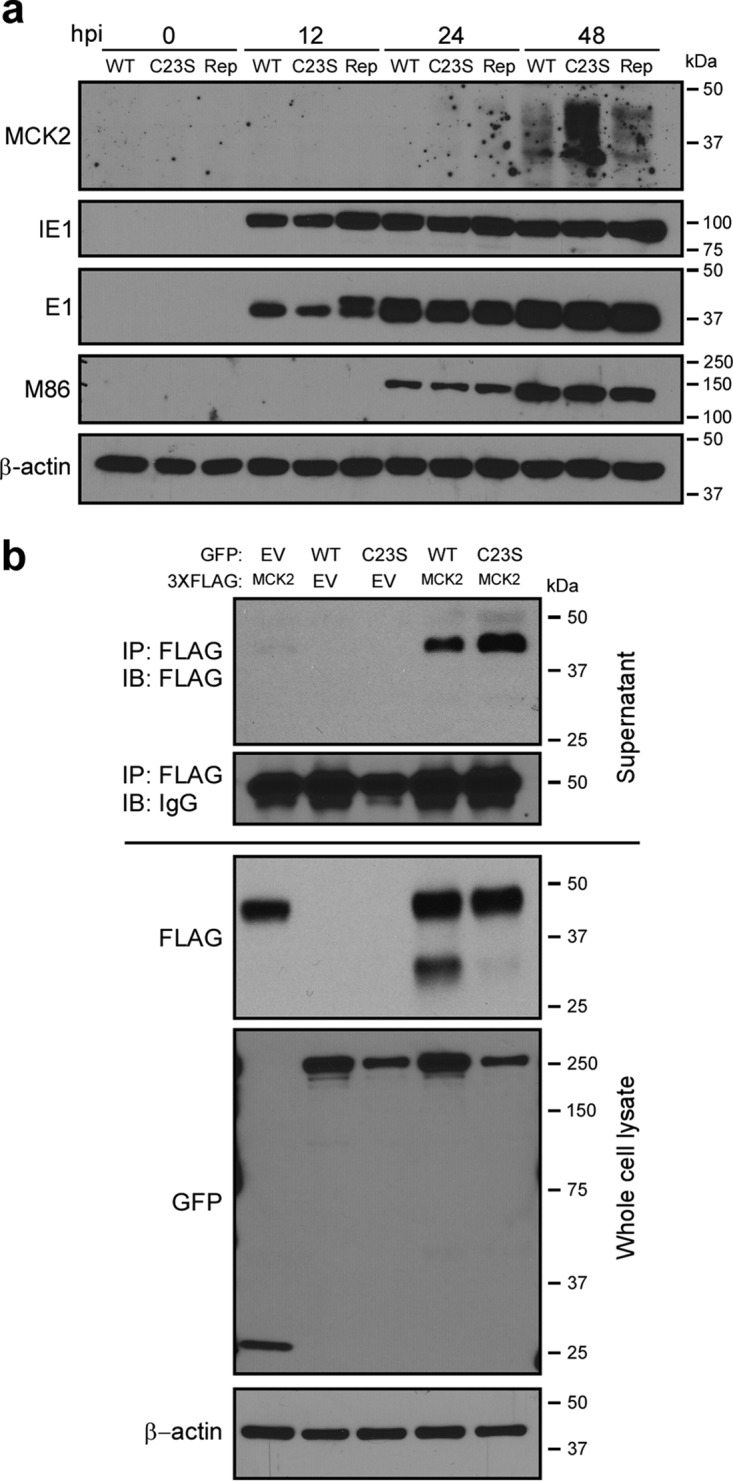
M48 DUB activity regulates MCK2 levels and can regulate secretion. (a) Immunoblot of whole-cell lysates from NIH3T3 fibroblasts infected with WT MCMV, MCMV-M48^C23S^, or MCMV-M48^Rep^ (MOI = 5.0). Samples were collected at the indicated times postinfection, and levels of MCK2, as well as those of representative immediate early (IE1), early (E1), and late [M86 (MCP)] viral antigens, were detected by immunoblotting. β-Actin served as a loading control. (b) Immunoblot (IB) analyses of supernatant immunoprecipitations (IP) (top panels) or whole-cell lysates (bottom panels) from NIH3T3 fibroblasts transfected with the indicated plasmids. At 24 h posttransfection, cell culture supernatants were collected and subjected to anti-FLAG immunoprecipitation as described in Materials and Methods. Cells were collected at the same time, and lysates were generated and proteins separated by SDS-PAGE. Samples were immunoblotted with the indicated antibodies. Data are representative of results from 3 independent experiments.

As MCMV-induced swelling is influenced by the production of the MCMV-encoded C-C chemokine, MCK2 (28–30), we next investigated what role the DUB activity of M48 may play in regulating this chemokine. As swelling occurred rapidly in the footpads of MCMV-M48^C23S^-infected mice, we hypothesized that MCK2 levels may be increased in MCMV-M48^C23S^-infected cells. In order to test this, NIH3T3 fibroblasts were infected (multiplicity of infection [MOI] = 5.0) and lysates collected at 0, 12, 24, and 48 hours post infection (hpi). An immunoblot assessment of MCK2 protein levels in WT-, MCMV-M48^C23S^-, or MCMV-M48^Rep^-infected fibroblasts revealed elevated MCK2 levels in MCMV-M48^C23S^-infected fibroblasts compared to the WT-infected or MCMV-M48^Rep^-infected fibroblasts (Fig. 3a). The levels of other representative immediate-early (IE1), early (E1), and late [M86/major capsid protein (MCP)] viral gene products were similar between the viruses during infection. These results suggest that M48 DUB activity negatively regulates MCK2 levels during infection.

To further test this hypothesis and assess the potential modulation of MCK2 by M48 DUB activity, M48 (WT or C23S) and MCK2 were cloned into the expression vectors pEGFP-N1 and p3XFLAG-CMV-14, respectively, and cotransfected into NIH3T3 murine fibroblasts. To directly address the effect of M48 DUB activity in regulating MCK2 secretion in the absence of infection, MCK2 was immunoprecipitated (IP) from the supernatants of cells cotransfected with epitope-tagged MCK2 and enhanced green fluorescent protein (EGFP)-tagged WT M48 or M48^C23S^ expression constructs. While MCK2 exhibited low levels of secretion in samples transfected with MCK2 alone, cotransfection with either M48 or M48^C23S^ showed elevated levels of MCK2 in the supernatant (Fig. 3b). Interestingly, MCK2 secretion was reproducibly higher in cells cotransfected with M48^C23S^, consistent with a model in which the M48 gene product promotes secretion of MCK2, and the DUB activity of M48 negatively regulates this process. Immunoblot analysis of cell lysates coexpressing MCK2 and WT M48, but not M48^C23S^ or EGFP alone, showed marked accumulation of an ~30-kDa band corresponding to the unglycosylated form of MCK2 (reference 31 ; see also Fig. S2a), suggesting that M48 DUB activity stabilizes or promotes accumulation of unglycosylated MCK2. This accumulation may result from a block of proteosomal degradation of MCK2, as evidenced by the accumulation of the unglycosylated 30-kDa band following a cycloheximide chase with MG132 pretreatment (Fig. S2b). These results suggest that MCK2 expression may be controlled by endoplasmic reticulum-associated protein degradation (ERAD) and that M48 DUB activity prevents MCK2 proteasomal degradation after retrotranslocation from the ER. An important constituent of the ERAD process is the AAA ATPase, p97/VCP, which extracts proteins from the ER in an ATP-dependent fashion and facilitates their ultimate degradation by the proteasome (32). Overexpression of a dominant-negative p97 (p97^QQ^) blocks retrotranslocation of proteins, such as the Rem signal peptide of mouse mammary tumor virus (MMTV), from the ER (33). Consistent with a role for ERAD in regulating MCK2 expression, cells cotransfected with p97^QQ^ showed a significant increase in MCK2 secretion (Fig. S2c). This result is similar to the observed increase in MCK2 secretion in the presence of M48, regardless of the presence or absence of DUB activity (Fig. 3b). Together, these results (Fig. 3 and S2) show that M48 plays DUB-dependent and -independent roles in the control of MCK2. Furthermore, they suggest that M48 may modulate ERAD or ERAD substrates like MCK2 to influence expression and secretion from infected cells and that this process is subject to fine control by M48 DUB activity.

10.1128/mBio.01864-16.2 FIG S2  MCK2 levels and secretion are controlled by ERAD. (a) NIH3T3 cells were transfected with EGFP, M48, or M48^C23S^ and with p3XFLAG–CMV-14 (EV) or MCK2. Immunoblot analysis was performed on whole-cell lysates (both untreated and treated with PNGase F). (b) NIH3T3 cells were transfected with MCK2 and then subjected to pretreatment with either dimethyl sulfoxide (DMSO) or MG132 (20 μM) 24 h posttransfection for 1 h prior to the addition of cycloheximide (CHX) (100 μg/ml). Whole-cell lysates were collected at 0, 4, 8, 12, and 24 h post-CHX treatment, and MCK2 was subjected to immunoblot analysis. *, unglycosylated MCK2 product. (c) NIH3T3 cells were transfected with MCK2 and pcDNA3.1 empty vector (EV), 6-His p97^WT^, or 6-His p97^QQ^. Secreted MCK2 was assessed as described for Fig. 3b. Download FIG S2, PDF file, 0.5 MB. Copyright © 2017 Hilterbrand et al. 2017 Hilterbrand et al. This content is distributed under the terms of the Creative Commons Attribution 4.0 International license.

### M48 DUB activity controls MCK2 incorporation into mature virions.

**FIG 4:**
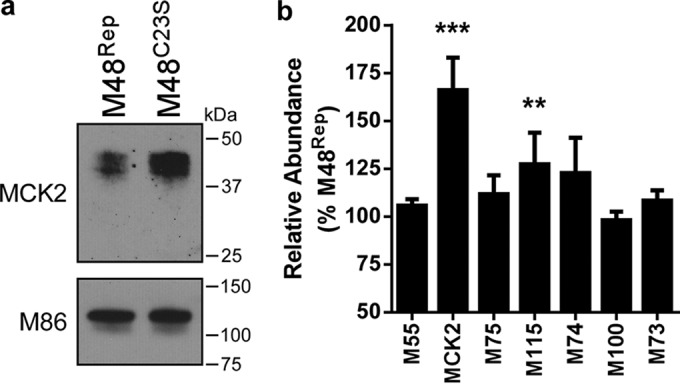
M48 DUB activity controls MCK2 incorporation into mature virions. (a) Immunoblot of lysates of MCMV-M48^Rep^ and MCMV-M48^C23S^ virions purified over a 20% to 70% linear sorbitol gradient. Equal amounts of virion lysate were loaded, and MCK2 levels were assessed by immunoblotting. M86 (MCP) served as a loading control between samples. (b) Relative peptide abundances of virion-associated glycoproteins M55 (gB), MCK2, M75 (gH), M115 (gL), M74 (gO), M100 (gM), and M73 (gN) in purified MCMV-M48^C23S^ virions compared to MCMV-M48^Rep^. Ratios were calculated as averages of target ion peak areas normalized to M86 (MCP) for three independent experiments. Error bars represent standard errors of the means. **, *P* < 0.01; ***, *P* < 0.001 (by Wilcoxon signed-rank test).

In addition to serving as a chemokine during infection, MCK2 can also associate with the glycoprotein H/glycoprotein L (gH/gL) complex in mature virions. gH/gL/MCK2 glycoprotein complexes promote pH- and energy-dependent entry and facilitate entry into macrophages (34). To determine if M48 DUB activity had an effect on virion-associated MCK2 levels, cell-free virions were purified by gradient centrifugation and subjected to immunoblot analysis. MCMV-M48^C23S^ virions contained higher MCK2 levels than MCMV-M48^Rep^ virions, while M86 (MCP) levels remained constant between samples (Fig. 4a). Mass spectrometry (MS)-based proteomics analysis of purified virions confirmed these findings. Label-free relative quantitation consistently showed higher levels of MCK2 protein in MCMV-M48^C23S^ virions than in MCMV-M48^Rep^ virions (Fig. 4b), indicating that, in addition to controlling MCK2 levels during infection, M48 DUB activity controls the amount of MCK2 incorporated into infectious virions. Interestingly, the MCK2 partner M115 (gL) also showed a modest and yet statistically significant increase in abundance, suggesting that MCK2 and gL may share a pathway for maturation and assembly regulated by the DUB. In contrast, no changes were observed in the relative levels of the major fusogenic glycoprotein M55 (gB) or of the components of the gM/gN glycoprotein complex, M100 (gM) and M73 (gN) (35) (Fig. 4b).

### Deletion of MCK2 rescues MCMV-M48^C23S^ replication *in vitro* and *in vivo*.

**FIG 5:**
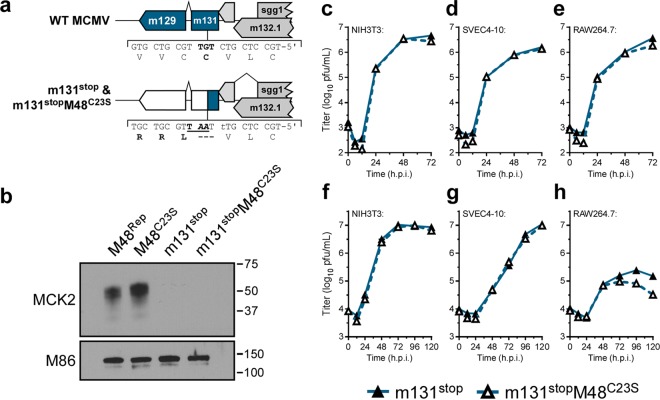
Concomitant loss of MCK2 rescues MCMV-M48^C23S^ replication in culture. (a) A schematic representing the genomic location and generation of mutant viruses lacking MCK2 expression (m131^stop^ or m131^stop^M48^C23S^). (b) Immunoblot of gradient-purified virions of the indicated genotype to confirm loss of MCK2 expression. (c to h) Single-step (c to e) (MOI = 5.0) and multistep (f to h) (MOI = 0.05) growth curves in NIH3T3 fibroblasts (c and f), SVEC4-10 endothelial cells (d and g), or RAW 264.7 macrophages (e and h). Each data point represents *n* = 3 biological replicates.

Given our results suggesting that M48 DUB activity controls MCK2 protein levels, we hypothesized that the MCMV-M48^C23S^ attenuation was at least partly due to the greater amounts of MCK2 being produced. If so, genetic ablation of MCK2 would restore replication of the DUB mutant virus. To directly test this hypothesis, two additional recombinant viral mutants were constructed. Both the WT and M48^C23S^ viruses were modified by introducing a translational stop and a single-nucleotide frameshift within the first exon (*m131*) of *Mck2*, generating MCMV-m131^stop^ and MCMV-m131^stop^M48^C23S^ (Fig. 5a and S3a, b, and c). In order to confirm a loss of MCK2 expression, gradient-purified virions were subjected to immunoblot analyses, with the results clearly demonstrating loss of MCK2 expression (Fig. 5b).

10.1128/mBio.01864-16.3 FIG S3  Generation of viruses lacking MCK2 expression. (a) Schematic diagram depicting the genomic area around MCK2 (m129-131) as well as the mutagenesis strategy to generate MCK2-deficient viruses. Numbers represent MCMV genomic coordinates (GenBank accession number AM886412.1), and abbreviations indicate restriction enzyme sites (Hi, HindIII; Ec, EcoRI; Ba, BamHI; Ms, MseI). Using a two-step allelic exchange strategy (see Materials and Methods), a selection/counterselection cassette was introduced into *m131* and was then replaced by a sequence introducing a single amino acid substitution and an additional nucleotide to generate a translational stop and a frameshift, which also introduces a unique MseI site. The nucleotides and amino acids that were changed are indicated in bold. Underlined sequences indicate an introduced restriction enzyme site. Insertion of the translational stop into m131 with either the WT BAC or M48^C23S^ was also verified by Sanger sequencing. The gray bar indicates the PCR amplicon generated as described for panel c. (b) Restriction fragment length polymorphism (RFLP) analysis of WT, m131SK, m131^stop^, M48^C23S^, m131SK M48^C23S^, and m131^stop^M48^C23S^ bacterial artificial chromosomes (BAC). Isolated DNAs were digested with EcoRI restriction enzyme, separated on 0.6% agarose gel, stained with ethidium bromide, and visualized on a Typhoon Imager. (c) Infectious virion DNA from the indicated viruses was isolated, and amplicons from the M48 locus (as described for Fig. S1c) or the m131 locus were generated by PCR. Amplicons were digested with BamHI or MseI to diagnose introduced mutations. Reactions were separated on 1.5% agarose gels, stained with ethidium bromide, and visualized on a Typhoon Imager. Download FIG S3, PDF file, 0.5 MB. Copyright © 2017 Hilterbrand et al. 2017 Hilterbrand et al. This content is distributed under the terms of the Creative Commons Attribution 4.0 International license.

Characterization of MCMV-m131^stop^ and MCMV-m131^stop^M48^C23S^ was initiated with single-step and multistep growth analyses in cell culture for NIH3T3 fibroblasts, SVEC4-10 endothelial cells, and RAW264.7 macrophages. Surprisingly, the single-step and multistep growth curves of MCMV-m131^stop^ and MCMV-m131^stop^M48^C23S^ showed no difference in replication kinetics in fibroblast and endothelial cell lines (Fig. 5c, d, f, and g), suggesting that the modest replication defect for MCMV-M48^C23S^ (Fig. 1b, c, e, and f) can be attributed to MCK2. Although MCMV-m131^stop^ and MCMV-m131^stop^M48^C23S^ replicated similarly in single-step replication analysis on RAW264.7 macrophages (Fig. 5e), MCMV-m131^stop^M48^C23S^ levels remained modestly attenuated during multistep replication (Fig. 5h). This result is similar to that shown in Fig. 1g, where MCMV-M48^C23S^ replicated at titers 4-fold to 5-fold lower than MCMV-M48^Rep^ or WT MCMV, suggesting that DUB-dependent functions may govern replication in macrophages independently of MCK2.

**FIG 6:**
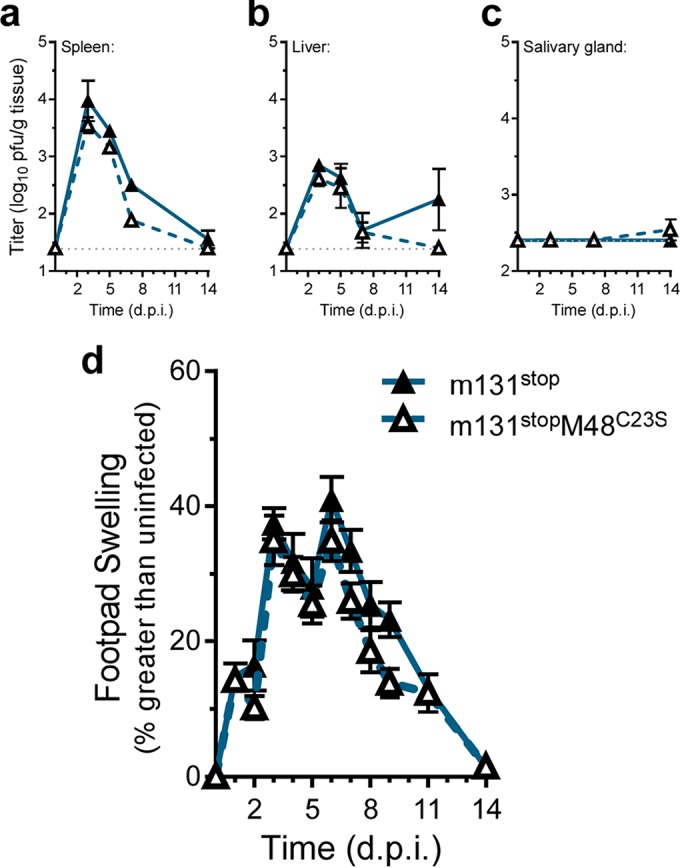
Concomitant loss of MCK2 in the presence of the DUB mutation rescues MCMV-M48^C23S^ replication in the spleens and livers and ameliorates footpad swelling. (a to c) C57BL6/J mice were infected with 10^6^ PFU of MCMV-m131^stop^ or MCMV-m131^stop^M48^C23S^ via i.p. injection. Viral load in the spleens (a), livers (b), and salivary glands (c) was assessed at the indicated times postinfection. Each data point represents *n* of ≥5 mice. (d) Footpad swelling of C57BL6/J mice infected with 10^6^ PFU of either MCMV-m131^stop^ or MCMV-m131^stop^M48^C23S^ via footpad injection. Swelling was measured by digital caliper over the course of 14 days, and data are plotted as percent increase over the measurements from uninfected mice. Each data point represents *n* of ≥5 mice.

To further investigate the role of MCK2 dysregulation in MCMV-M48^C23S^ replication in a natural host, animals were infected with *Mck2*-deficient viruses by i.p. injection. Concomitant loss of MCK2 reversed the acute replication defect of MCMV-M48^C23S^ to WT levels in spleen and livers of infected mice over a time course of 14 days postinfection (Fig. 6a and b). *Mck2*-deficient viruses were not detected in salivary glands of infected mice (Fig. 6c), consistent with a role for MCK2 in virus dissemination. To determine if the rapid onset of footpad swelling was dependent on MCK2 expression, animals were infected via footpad injection with either MCMV-m131^stop^ or MCMV-m131^stop^M48^C23S^. Mice infected with either MCMV-m131^stop^ or MCMV-m131^stop^M48^C23S^ showed identical levels of swelling and recovery over the 14-day time course with no rapid onset of swelling (Fig. 6d) as seen in the MCMV-M48^C23S^-infected mice (Fig. 2d). This suggests that the DUB-dependent swelling phenotype observed in MCMV-M48^C23S^-infected mice (Fig. 2d) was mediated by MCK2. Taken together, these results indicate that M48 DUB activity regulates MCK2 biology by controlling its expression, maturation, or secretion during infection.

## DISCUSSION

In this report, we describe a systematic investigation into the role of the MCMV DUB *in vitro* and *in vivo*. Specific mutation of a catalytic cysteine within the DUB domain of M48 (C23S) revealed a minor role for M48 DUB activity in MCMV replication in cultured cells (see Fig. 1b to g) but substantial attenuation in a natural host (Fig. 2a to c). Importantly, these results are consistent with previous reports demonstrating that the DUB activity of the HCMV orthologue of M48, UL48, is present during infection and modestly contributes to replication in cell culture (17). Interestingly, our work revealed that a major role for M48 DUB activity is control of MCK2, the viral chemokine, both *in vitro* and *in vivo*. Loss of M48 DUB function results in increased MCK2 levels (Fig. 3a), manifesting as enhanced inflammation at localized sites of infection in mice (Fig. 2d) and resulting in attenuation of MCMV replication (Fig. 2a to c and 6a and b). Concomitant loss of MCK2 restored most observed DUB-dependent phenotypes *in vitro* and *in vivo* (Fig. 5 and 6), providing compelling genetic evidence for a mechanism of intrinsic control over the virus-encoded chemokine.

Accumulating evidence implicates cellular DUBs in the control of a number of host proinflammatory pathways. The host deubiquitinase, A20, is critical for regulating NF-κB activation and subsequent activation of proinflammatory cytokines (36, 37). Additionally, recent work has shown that the cellular DUB, Trabid, negatively regulates expression of interleukin-12 (IL-12) and IL-23 via the modification of ubiquitinated histones (38). OTULIN, the Met1-linked deubiquitinase, controls levels of Met1-linked ubiquitin chains, modulating activation of NF-κB responses and TNF-α levels (39). While herpesvirus DUBs have been implicated in the control of host inflammatory signal transduction (19–21), M48 appears unique in that it manages levels of MCK2, a virally encoded chemokine. While our results do not exclude the possibility that M48 DUB activity may regulate other pathways, they provide the first example of a herpesvirus DUB controlling levels of a viral chemokine.

In order to determine whether MCK2 production contributed to the observed phenotypes of the MCMV-M48^C23S^ virus, we took a genetic approach and ablated MCK2 expression. Surprisingly, concomitant loss of MCK2 in the presence of the mutant DUB restored replication in most cell types in culture (Fig. 5c to h). Fibroblasts and endothelial cells infected with MCMV-m131^stop^ or MCMV-m131^stop^M48^C23S^ displayed similar replication kinetics in single-step and multistep replication analyses. Although the mechanism of attenuation of MCMV-M48^C23S^ in fibroblast and endothelial cells is likely complex, recent findings suggest that increased levels of virion-associated MCK2 (Fig. 4a and b) promote an endocytic mode of entry over fusion at the plasma membrane (34). Endocytic entry of herpesviruses results in delayed gene expression and increased antiviral gene transcription (40), both of which could contribute to attenuation of virus replication. Increased levels of these antiviral proteins may occur in MCMV-M48^C23S^-infected cells, resulting in modest attenuation. We have recently shown that MCMV-M48^C23S^ elicited a greater stimulator of interferon gene (STING)-dependent type I IFN response in cultured dendritic cells (22). However, whether virion-associated MCK2 levels and STING-dependent responses during infection are connected remains to be determined.

Interestingly, while no difference in replication was observed in single-step growth kinetics on macrophages (Fig. 5e), loss of MCK2 in the presence of the mutant DUB failed to alleviate the modest growth defect in multistep growth (Fig. 5h), similarly to that seen in Fig. 1g. These results imply that DUB-dependent, MCK2-independent mechanisms may be necessary for efficient replication in macrophages. Since macrophages and monocytes are important CMV target cells *in vivo*, a potential role for DUB-dependent functions in cell tropism warrants further investigation.

Surprisingly, replication of MCMV-M48^C23S^ in the spleens and livers of infected mice was restored with targeted deletion of MCK2. Previous work examining the contributions of MCK2 to pathogenesis has shown that, while MCK2 is necessary for dissemination to the salivary glands, it is dispensable for replication and spread in the spleen and liver (29, 30, 41). Our results are consistent with these findings showing that MCK2 is not a major contributor to the early stages of infection in target organs. We extend these observations to show that overproduction and/or dysregulation of MCK2, as seen in MCMV-M48^C23S^-infected cells, attenuates acute replication *in vivo*.

MCK2 is necessary for effective dissemination of MCMV to the salivary glands of infected mice (29, 30, 42, 43). However, MCMV-M48^C23S^, which fails to appropriately control MCK2 production or secretion, remained impaired for dissemination to the salivary glands regardless of the route of inoculation (Fig. 6C and S4 in the supplemental material). Thus, it is tempting to speculate that careful calibration of MCK2 levels facilitates a successful infection. Recent work utilizing recombinant MCMVs expressing viral or mammalian neutrophil-attracting chemokines (mouse CXCL1 or HCMV CXCL1) showed that overexpression of proinflammatory chemokines is detrimental to MCMV dissemination to the salivary gland (44). Our work is consistent with those findings in that uncontrolled MCK2 levels impair dissemination of MCMV. However, as expected, neither the MCMV-m131^stop^ nor the -m131^stop^M48^C23S^ viruses were reliably detected in the salivary glands of infected mice (Fig. 6c), indicating that MCK2 is required for dissemination. Thus, it is difficult to conclude that MCK2 overproduction is the only contributor to a decrease in mutant DUB virus dissemination. DUB-dependent, MCK2-independent contributions to MCMV dissemination and/or salivary gland replication may exist. The modest replication defect seen in multistep replication kinetics in macrophages (Fig. 1g and 5h) could indicate the existence of DUB-dependent contributions to monocyte infection and dissemination independent of MCK2.

10.1128/mBio.01864-16.4 FIG S4  MCMV-M48^C23S^ fails to disseminate to salivary glands following footpad inoculation. Day 14 salivary glands titers from C57BL6/J mice infected with MCMV-M48^Rep^ or MCMV-M48^C23S^ (orange circles) and MCMV-m131^stop^ or MCMV-m131^stop^M48^C23S^ (blue triangles). Organs were harvested from animals used in the footpad swelling experiments described for Fig. 2d and 6d. Each point represents one animal. Data represent means ± standard errors of the means (SEM). **, *P* < 0.01. Download FIG S4, PDF file, 0.03 MB. Copyright © 2017 Hilterbrand et al. 2017 Hilterbrand et al. This content is distributed under the terms of the Creative Commons Attribution 4.0 International license.

In addition to attenuation of acute replication, MCMV-M48^C23S^ induced a robust inflammatory response following footpad inoculation. We showed that this phenotype is restored to WT levels by concomitant loss of MCK2 (Fig. 6d). This result clearly indicates that the rapid swelling observed in MCMV-M48^C23S^-infected mice was due to MCK2 overproduction. Unlike previous footpad studies performed with MCK2-null viruses (29, 30, 42, 43), we observed considerable swelling in the footpads of mice infected with MCMV-m131^stop^ or MCMV-m131^stop^M48^C23S^. This inconsistency may be due to several factors. In previous works, the K181+ strain and its MCK2 mutant derivatives were used, while our studies utilized the BAC-derived K181 Perth strain (pARK25) of MCMV. Additionally, most studies of MCK2-dependent inflammation have employed BALB/c mice, whereas we report results from C57BL/6 mice. While the source of these differences in the results of control experiments warrants further investigation, we clearly demonstrated that the rapid swelling seen in MCMV-M48^C23S^-infected mice is ameliorated by ablating MCK2 expression. This is compelling evidence that loss of DUB control over MCK2 expression, maturation, or secretion mediates the inflammatory phenotype of MCMV-M48^C23S^ *in vivo*.

Although the specific substrate(s) of M48 DUB activity during infection remains unknown, we anticipate that significant targets include endoplasmic reticulum- and/or Golgi-associated components. Interestingly, the M48 homologue encoded by HCMV, UL48, has been shown to interact with the endoplasmic reticulum protein RRBP1/ES130 (45), a ribosome binding protein critically implicated in the function of the secretory pathway (46, 47). Although the functional relevance was not addressed, considered together with the data reported here, a possible model emerges wherein betaherpesvirus DUBs may be localized to the ER, allowing control over secreted or transmembrane protein maturation, whether host or virus in nature. This association at the ER might allow M48 to affect secretory events via RRBP1 by modulating ERAD (Fig. S2) or through another currently unknown mechanism. Therefore, the identification of cellular binding partners and substrates of MCMV M48 will be imperative for understanding how CMV DUB activity controls these processes.

In conclusion, we have revealed a novel mechanism by which a herpesvirus DUB controls the host response to infection and appropriates that response for successful infection. While the function of the viral chemokine MCK2 in eliciting and coopting host inflammatory responses has been previously described (28–30, 42, 43), we show that there is DUB-dependent control of MCK2 expression during infection. Thus, a unique interplay between M48 DUB activity and MCK2 function orchestrates a critical step in CMV pathogenesis, representing a potential target for therapeutic intervention.

## MATERIALS AND METHODS

### Plasmids and transfections.

Transfections were performed with GenJet *in vitro* transfection reagent (Ver. II; SignaGen Laboratories) per the manufacturer’s instructions. Carboxy-terminal 3XFLAG epitope-tagged MCK2 was generated by PCR amplification of nucleotides 188352 to 187431 from pARK25 (48), a bacterial artificial chromosome (BAC) containing the K181 (Perth) strain MCMV (GenBank accession number AM886412.1). Amplicons were cloned into the *EcoRI* and *XbaI* sites of p3XFLAG–CMV-14 (Sigma-Aldrich). An untagged MCK2 expression construct was similarly generated by PCR and the resulting amplicon cloned into the *KpnI* and *BamHI* sites of pcDNA3.1(+). Carboxy-terminal EGFP-tagged M48 and M48^C23S^ were generated by PCR amplification of nucleotides 67043 to 67897 (amino acids [a.a.] 1 to 285) or 67043 to 73483 (full length) of either WT BAC or M48^C23S^ BAC, respectively. Both the full-length and M48^(1–285)^ constructs were cloned into the *HindIII* and *KpnI* sites of pEGFP-N1 (Clontech). Plasmids pQE9-His-p97(wt) and pQE9-p97(QQ) (Addgene plasmids 14666 and 14667, respectively) were a gift from Graham Warren (49, 50). Insertions were amplified by PCR with an N-terminal 6-His tag and cloned into the *KpnI* and *BamHI* sites of pcDNA3.1(+).

### Cells.

NIH3T3 murine fibroblasts (ATCC CRL-1658) were propagated in Dulbecco’s modified Eagle’s medium (DMEM; Sigma-Aldrich) containing 10% heat-inactivated bovine calf serum (BCS; Life Technologies, Inc.) and 1% penicillin-streptomycin-glutamine (Life Technologies, Inc.). RAW264.7 murine macrophages (ATCC TIB-71) and SVEC4-10 endothelial cells (ATCC CRL-2181) were propagated in DMEM (Sigma) containing 10% heat-inactivated fetal calf serum (Life Technologies, Inc.) and 1% penicillin-streptomycin-glutamine (Life Technologies, Inc.).

### BAC mutagenesis and recombinant viruses.

pSIM6 plasmid, containing genes necessary for λ-red recombination, was introduced into bacteria containing pARK25 (48). Recombineering for K181-BAC mutagenesis was performed as previously described (51). Genome integrity was confirmed by restriction fragment length polymorphism analysis, and mutagenesis was verified by PCR, restriction digest, and sequencing of the targeted region of *M48* and *m131*. BAC-derived parental and recombinant viruses were generated and purified as previously described (48). Viruses were propagated, clarified, and concentrated, and the titers were determined by plaque assay on NIH3T3 cells as previously described (30). Growth curve experiments were performed in 12-well plates at the indicated multiplicity of infection (MOI) in 0.4 ml for 2 h at 37°C. After adsorption, cells infected at an MOI of 5.0 were washed twice with phosphate-buffered saline (PBS) and refed. Cells infected at an MOI of 0.05 in a volume of 0.4 ml were given additional complete DMEM to reach a volume of 1 ml. Cells and supernatants were harvested at the indicated times, and titers were determined via plaque assay on NIH3T3 fibroblasts.

### Immunoprecipitation and immunoblotting.

Culture supernatants collected from MCK2/M48-cotransfected cells were cleared of cell debris by spinning at 5,000 × *g* for 15 min. IPs were performed by adding anti-FLAG M2-agarose slurry (Sigma-Aldrich) followed by incubation on an orbital rotator overnight at 4°C. Cell lysates and IP samples were separated by SDS-PAGE on 10% acrylamide gels or Mini-Protean TGX precase 4% to 20% gradient gels (Bio-Rad). Proteins were transferred to nitrocellulose membranes (GE Healthcare Life Sciences) and subjected to immunoblot analysis with indicated antibodies. The following antibodies were used in immunoblot analyses: mouse anti-MCK2 (clone 11D7; gift from Peggy MacDonald, the Rockefeller University), mouse anti-m123/IE1 (Chroma101; Center for proteomics, University of Rijeka), mouse anti-m112-113/E1 (Chroma103; Center for proteomics, University of Rijeka), rabbit anti-M86 (MCP) (gift from Laura Hanson, Texas Woman’s University), mouse anti-β-actin (clone AC-74; Sigma-Aldrich), mouse anti-GFP (clone 4B10; Cell Signaling Technology, Inc.), mouse anti-FLAG M2-peroxidase (clone M2; Sigma-Aldrich), mouse anti-ubiquitin (clone P4D1; Santa Cruz), mouse anti-6-His (THE His Tag antibody; GenScript), anti-mouse IgG-horseradish peroxidase (IgG-HRP) (Vector Laboratories), and anti-rabbit IgG-HRP (Vector Laboratories).

### PNGase F treatment.

PNGase F treatments were performed according to the protocol of the manufacturer (NEB) for denaturing conditions. Briefly, whole-cell lysate in NP-40 lysis buffer (1% NP-40, 150 mM NaCl, 50 mM Tris-HCl—pH 8.0) was diluted with denaturing buffer and boiled for 10 min. Reaction buffer (10×; diluted to 1×), NP-40 (1%), and PNGase F were added, and the reaction mixture was incubated at 37°C for 1 h. Samples were then analyzed by immunoblotting.

### Virus purification.

Virions were purified over a 20% to 70% linear sorbitol gradient as described previously (52). Briefly, extracellular virions were collected from infected cell supernatants via centrifugation at 20,000 × *g* for 1.5 h at 4°C. Virion pellets were collected and then passed over a 20% sucrose cushion for 1 h at 16°C and 32,800 × *g*. The cushion-purified virus was resuspended in 1 ml of TN buffer (50 mM Tris-HCl and 100 mM NaCl, pH 7.4), briefly sonicated, and placed on the top of the linear gradient. Gradients were centrifuged at 76,000 × *g* in an SW41 swinging-bucket rotor for 1 h at 16°C. Virion-containing bands were collected by aspiration, diluted in buffer, and pelleted by centrifugation at 100,000 × *g* for 3 h at 16°C.

### Sample preparation for LC-MS/MS analysis.

Pelleted virions were resuspended in TN buffer, and 2,2,2-trifluoroethanol (TFE) (Sigma-Aldrich) was added to reach a final concentration of 50% TFE. Proteins were reduced with 5 mM tris(2-carboxyethyl)phosphine (TCEP) for 45 min at 55°C followed by alkylation with 15 mM chloroacetamide at room temperature, in the dark, for 30 min. Excess chloroacetamide was quenched with 20 mM dithiothreitol (DTT), and samples were diluted 10-fold to reach a final concentration of 5% TFE–digestion buffer (50 mM Tris [pH 8.0], 2 mM CaCl_2_). Trypsin was added to reach a final concentration of 1:50 (enzyme/protein), and the digests were incubated at 37°C for 5 h. The digests were quenched by addition of formic acid to reach a final concentration of 1%. Tryptic digests were vacuum centrifuged to reduce the volume to approximately 100 to 150 μl. Buffer exchange was performed with Hypersep SpinTip C18 SPE tips (Thermo Scientific) according to the manufacturer instructions. Briefly, resin was washed 3 times with a 60% acetonitrile–0.1% formic acid solution and then equilibrated with buffer A (0.1% formic acid–water). Peptides were passed over the resin, after which bound peptides were washed 3 times with buffer A and eluted with the 60% acetonitrile–0.1% formic acid solution. Eluted peptides were briefly dried via vacuum centrifugation and resuspended in 5% acetonitrile–0.1% formic acid. Peptides were stored at −80°C until liquid chromatography-tandem mass spectrometry (LC-MS/MS) analysis.

### LC-MS/MS analysis.

Samples were analyzed by liquid chromatography-tandem mass spectrometry (LC-MS/MS), with a minimum of three replicate injections analyzed per sample. Peptides were separated by reverse-phase chromatography on a Dionex Ultimate 3000 nanoRSLC system (Thermo Scientific) with an Acclaim PepMap 100 RSLC C_18_ column (Thermo Scientific) (15 cm), using an acetonitrile gradient (3% to 38% over 215 min). Eluting peptides were directly analyzed by nano-electrospray ionization-tandem mass spectrometry on an Orbitrap Velos Pro mass spectrometer (Thermo Scientific). Full spectra (MS1) were collected at a resolution of 100,000. Fragmentation spectra (MS2) were collected in a data-dependent manner, with ions required to carry a charge of +2 or greater for MS2 selection, and up to 20 MS2 scans were collected per round. Dynamic exclusion was employed, whereby ions selected twice within 30 s were excluded from selection for 45 s.

### MS data analysis and protein quantitation.

A searchable protein sequence database was constructed from MCMV (strain K181) and mouse reference proteomes (UniProt) and common contaminants (from the MaxQuant website; http://maxquant.org/downloads.htm). MS spectra were searched against this database using SEQUEST (Proteome Discoverer 1.4; Thermo Scientific). The database was curated to include the m129/m131 spliced product. Fully tryptic peptides with up to 2 missed cleavages were considered. Mass tolerance filters of 10 ppm (MS1) and 0.5 Da (MS2) were applied. Modifications of cysteine carbamidomethylation (static; +57.0215 Da) and methionine oxidation (dynamic; +15.9949 Da) were allowed, with no more than three modifications allowed per peptide-spectrum match (PSM). False-discovery rates (FDR) for PSMs were determined by decoy database error modeling using Percolator (Proteome Discoverer 1.4; Thermo Scientific), and a set of high-confidence PSMs were selected for further analysis at an FDR of <1%. Extracted-ion chromatograms (XICs) of MCMV-derived peptides were manually inspected using the Skyline software package (53) to identify target ions exhibiting minimal interference and limited variability across replicate injections. The top three highest-quality target ions were selected for each protein of interest and XIC peak areas exported for calculation of relative abundances. XIC peak areas were first normalized to the major capsid protein M86 (MCP) by dividing XIC peak areas by the mean of the values determined for the three M86 (MCP) target ions. Normalized values were averaged across replicate injections and used to calculate the target ion ratios between M48^C23S^ and M48^Rep^ samples. Finally, mean averages and standard deviations of target ion ratios were calculated for each protein.

### Animal experiments.

C57BL/6J mice were obtained from the Jackson Laboratory. Animals were bred and maintained at the Animal Resources Center (ARC) at the University of Texas at Austin in accordance with Institutional guidelines, and all procedures were approved by the University of Texas at Austin Institutional Animal Care and Use Committee. Six- to 10-week-old male and female animals were infected with 10^6^ PFU by inoculation into a rear footpad or by intraperitoneal (i.p.) injection, as previously described (51). Upon sacrifice, organs were placed into 1 ml of complete DMEM, subjected to a single freeze/thaw cycle (−80°C), and disrupted by sonication. Organ homogenates were serially diluted, and the titers were determined by plaque assay on NIH3T3 fibroblasts. Footpad measurements were taken at the indicated time points with a digital caliper, as previously described (29). Unless otherwise indicated, each time point represents *n* of ≥5.
