# Supplementary figures and images for: Murine Cytomegalovirus Deubiquitinase Regulates Viral Chemokine Levels To Control Inflammation and Pathogenesis
Source: mBio. 2017 Jan 17;8(1):e01864-16. doi: 10.1128/mBio.01864-16 (PMC5241396; doi:10.1128/mBio.01864-16)

**Figure S1**

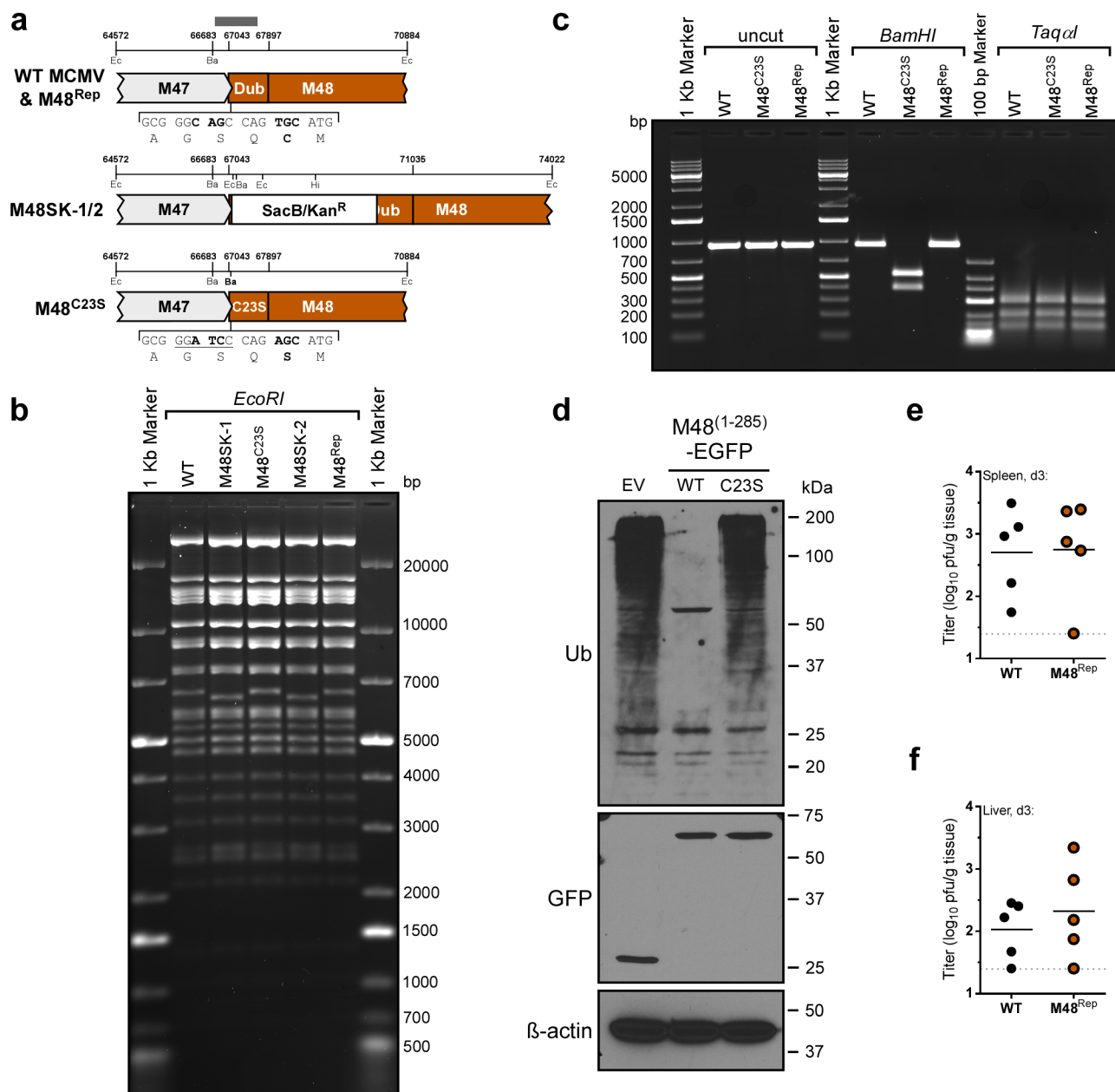

Supplement: FIG S1 [file mbo002173151sf1.pdf]

Figure S2

a

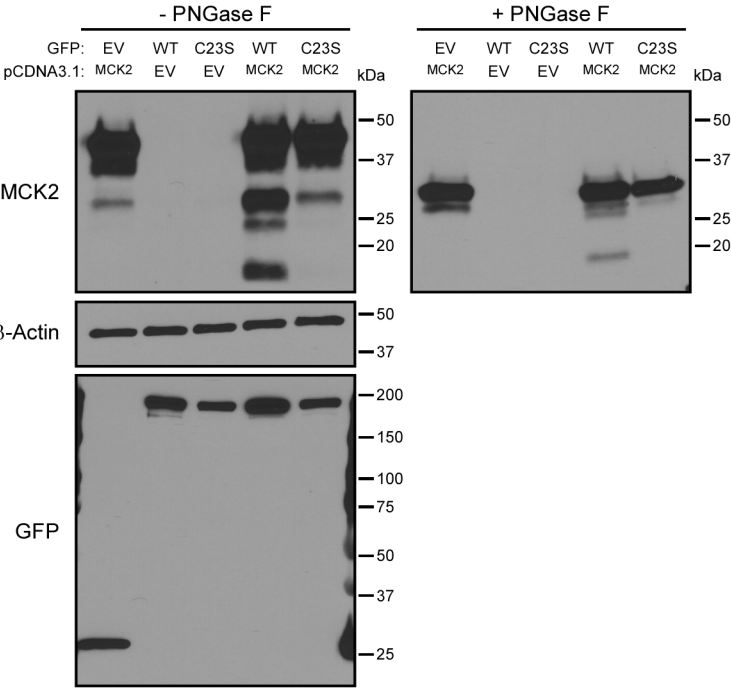

b

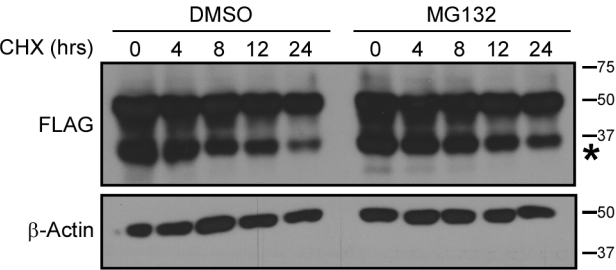

c

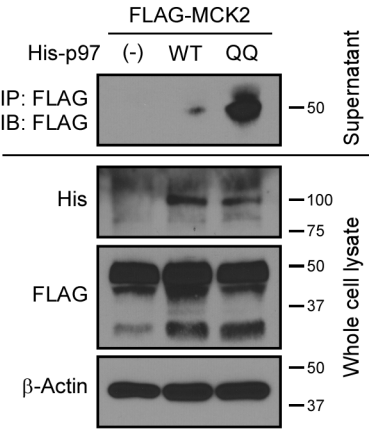

Supplement: FIG S2 [file mbo002173151sf2.pdf]

**Figure S3**

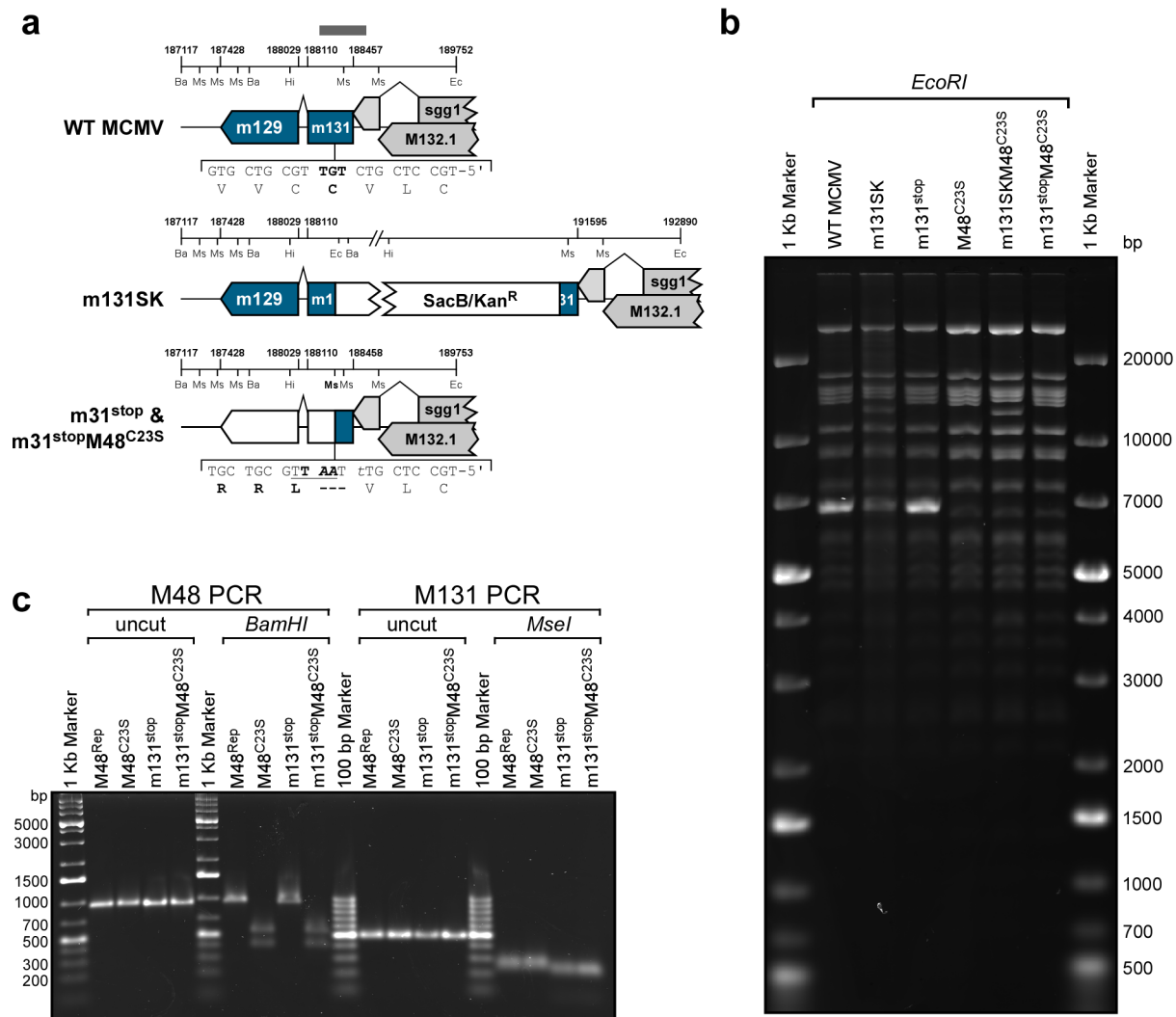

Supplement: FIG S3 [file mbo002173151sf3.pdf]

Figure S4

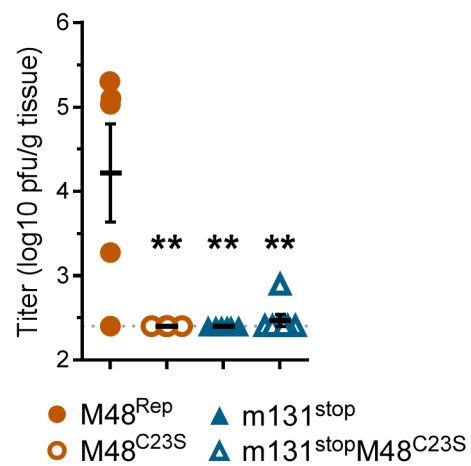

Supplement: FIG S4 [file mbo002173151sf4.pdf]
